# An ambient-temperature stable nanoparticle-based vaccine for nasal application that confers long-lasting immunogenicity to carried antigens

**DOI:** 10.3389/fimmu.2022.1057499

**Published:** 2022-10-31

**Authors:** Adolfo Cruz-Resendiz, Gonzalo Acero, Alicia Sampieri, Goar Gevorkian, Carolina Salvador, Laura Escobar, Margarita Jacaranda Rosendo-Pineda, Mara Medeiros, Luis Vaca

**Affiliations:** ^1^ Instituto de Fisiología Celular, Universidad Nacional Autónoma de México. Ciudad Universitaria, Mexico City, Mexico; ^2^ Departamento de Microbiología y Parasitología, Facultad de Medicina, Universidad Nacional Autónoma de México. Ciudad Universitaria, Mexico City, Mexico; ^3^ Instituto de Investigaciones Biomédicas, Universidad Nacional Autónoma de México. Ciudad Universitaria, Mexico City, Mexico; ^4^ Departamento de Fisiología, Facultad de Medicina, Universidad Nacional Autónoma de México. Ciudad Universitaria, Mexico City, Mexico; ^5^ Nephrology Research Laboratory, Hospital Infantil de México Federico Gómez, Mexico City, Mexico

**Keywords:** polyhedrin, baculovirus, occlusion bodies, nasal immunization, nanoparticles

## Abstract

Polyhedrins are viral proteins present in a large family of baculoviruses that form occlusion bodies (polyhedra). These structures protect the virus particles from the outside environment until they are ingested by susceptible insects. Occluded viruses can sustain inclement weather for long periods of time. Therefore, the polyhedra is a natural preservative that keeps the viral structure intact at ambient temperature for years. In a previous study we identified the first 110 amino acids from polyhedrin (PH_(1-110)_) as a good candidate to carry antigens of interest. As a proof of concept, we produced a fusion protein with PH_(1-110)_ and the green fluorescent protein (PH_(1-110)_GFP). The fusion protein associates spontaneously during its synthesis resulting in the formation of nanoparticles. Nasal immunization with these nanoparticles and in the absence of any adjuvant, results in a robust immune response with the production of IgG immunoglobulins that remained elevated for months and that selectively recognize the GFP but not PH_(1-110)_. These results indicate that PH_(1-110)_ is poorly immunogenic but capable of enhancing the immune response to GFP.

## Introduction

Baculoviruses (family Baculoviridae) are a group of DNA viruses that infect a wide variety of insects (1). They receive their name due to the rod shape (baculo) of the virus when observed under electron microscopy (2). In addition to the core genes found in all baculoviruses, lepidopteran baculoviruses encode an additional set of genes including a gene producing polyhedrin, the main component forming the occlusion bodies known as polyhedra (3, 4). Occluded viruses can withstand the inclement weather for years, retaining their infectivity, while unoccluded viruses become unviable within a few hours at ambient temperature. For this reason, occluded bodies are considered as preservative reservoirs, keeping the virus viable under harsh environmental conditions that otherwise would inactivate them.

Several studies have utilized the display of antigens on the surface of free (unoccluded) baculoviruses to produce vaccines. Immunization of mice with baculoviruses carrying antigens of interest on their surface result in the production of antibodies that recognize the original pathogen (5–7). Several pathogen proteins have been display on the surface of baculoviruses with promising results, including Varicela-Zoster (8), Toxoplasma gondii (9), influenza (10) SARS-Cov (11) and more recently SARS-Cov-2 (12).

The fusion of antigens from baculovirus to the occlusion bodies have also been attempted. Several antigens such as herpesvirus II (13), *Mycobacterium bovis* (14), porcine circovirus type 2 (15), foot-and-mouth disease virus (16), and many others (17) have been fused to the wild type polyhedrin gene to generate recombinant occlusion bodies. Mice immunized with the recombinant polyhedras produced antibodies that recognize the pathogen, and in some studies prevent the disease (15, 18).

Thus, baculovirus and occlusion bodies display are two powerful tools with feasible applications in the generation of novel vaccines. However, we know little about the immunogenicity of the polyhedrin alone since studies have been focused on the immunogenicity of the recombinant fusion protein (polyhedrin+antigen). Furthermore, we have explored several fragments of the polyhedrin to determine which of them can form occlusion-like aggregates, to reduce the size of the polyhedrin sequence utilized as carrier (19). Reducing the size of the carrier protein may reduce or prevent the deviation of the immune response, which could result in the generation of a robust immune response against the carrier but not the antigen of interest. Another problem which may result from the use of highly immunogenic carriers is immune tolerance (20–22). This phenomenon can be especially problematic when multiple vaccine doses are required after several immunizations with different antigens using the same carrier.

In the present study we aimed to explore the use of a fragment from polyhedrin (the first 110 amino acids, PH_(1-110)_) as a novel carrier for nasal vaccination. Here we show that PH_(1-110)_ spontaneously aggregates into nanoparticles (NPs) that can be easily purified by low-speed centrifugation as previously reported (19, 23). Nasal immunization in mice with PH_(1-110)_ carrying the green fluorescent protein (GFP) results in the production of circulating IgA, IgG and IgM anti-GFP antibodies in the serum of the subjects and in bronchoalveolar lavage fluid (BALF). Most notably, we could not detect antibodies against PH_(1-110)_, indicating that this fragment of polyhedrin is poorly immunogenic. Also worth highlighting is the fact that the PH_(1-110)_GFP NPs required no adjuvant to induce a robust immune response that lasted for more than six months. On the other hand, GFP alone (not fused to PH_(1-110)_) required the use of an adjuvant to induce an immune response with lower antibody titers compared to PH_(1-110)_GFP.

All these results position the fragment PH_(1-110)_ from polyhedrin as a viable carrier for antigen production, purification, and delivery of novel particulate vaccines for mucosal immunization.

## Material and methods

### Ethics statements

All study procedures were approved by the Internal Committee for the Care and Use of Laboratory Animals (CICUAL) of the Institute of Cellular Physiology (Protocol number LVD164-20), National Autonomous University of Mexico (UNAM). The care, feeding, and handling of the animals were carried out in strict compliance with the guidelines established by the Official Mexican Standard NOM-062-ZOO-1999. All animals were kept in a pathogen-free environment. *Ad libitum* feed and water, sterile sawdust bedding, and regular cleaning were provided.

### Recombinant protein design

**Figure 1:**
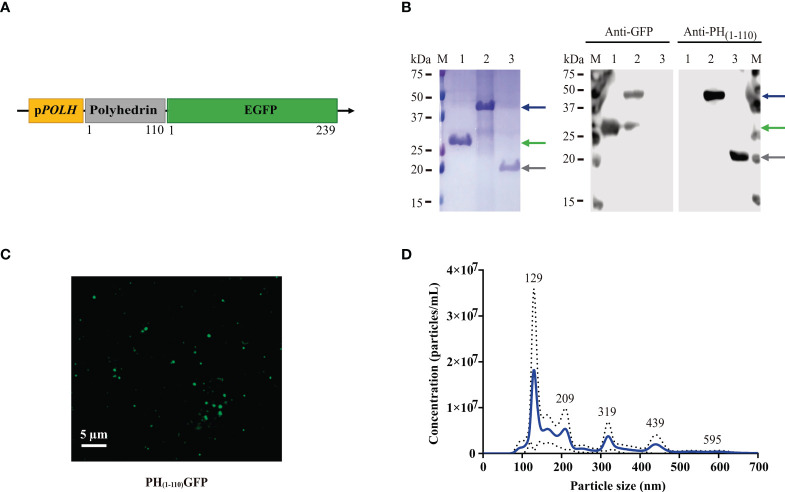
Generation and characterization of PH_(1-110)_GFP NPs. **(A)** Schematic diagram of the genetic construct for the expression of PH_(1-110)_GFP NPs in the baculovirus insect cell expression system. The coding sequences for the N-terminal 110 aa of polyhedrin and GFP were inserted under the pPOLH promoter. **(B)** SDS-PAGE and Western Blot analysis of PH_(1-110)_GFP expression. Lane M: molecular weight markers; Lane 1: GFP; Lane 2: PH_(1-110)_GFP; Lane 3: PH_(1-110)_. Blue arrow: PH_(1-110)_GFP (~42 kDa); Green arrow: GFP (~28 kDa); Gray arrow: PH_(1-110)_ (~19 kDa). **(C)** Confocal microscopy of purified PH_(1-110)_GFP NPs. **(D)** Nanoparticle Tracking Analysis of purified PH_(1-110)_GFP NPs. Data represent mean ± SD.

The construction of the fusion protein PH_(1-110)_GFP (**Figure 1A**) was designed and previously published by our laboratory (23). Briefly, the expression vector pFastbacTM1 of the Bac-to-Bac^®^ baculovirus expression system (Thermo Fisher, USA, cat. no. 10359-016) was used for cloning. The 1-110 aa N-terminal coding sequence of polyhedrin (PH_(1-110)_) was cloned under the polyhedrin (POLH) promoter. The green fluorescent protein (GFP) sequence (GenBank: AAB08058.1) was ligated at the 3’ end of the PH_(1-110)_ sequence. For amplification and titration of the viruses, the manufacturer’s recommendations were followed.

The GFP coding sequence was cloned in open reading frame into the pET-28a(+) vector (Sigma-Aldrich, USA cat. no. 70777) for expression in a bacterial system. The construct added an N-terminal His-tagged and the Kanamycin resistance gene. The same procedure was performed to clone the PH_(1-110)_ sequence into the pET-28a(+) vector (Sigma-Aldrich, USA cat. no. 70777).

### Cell line and bacterial strains

Spodoptera frugiperda cell line, Sf9 (ATCC^®^, USA cat. no. CRL-1711), was used for the propagation and titration of recombinant baculoviruses, later they were also used for the expression of PH_(1-110)_GFP nanoparticles (NPs) (19). Cells were maintained in Grace medium (Thermo Fisher, USA, cat. no. 11300-027) supplemented with 10% inactivated fetal bovine serum (FBS) (Biowest, France, cat. no. S1650-500), lactalbumin (Sigma -Aldrich, USA, cat. no. 19010), yeastolate (Thermo Fisher, USA, cat. no. 292805), antibiotic-antimycotic (Thermo Fisher, USA, cat. no. 15240-062), and 0.1% pluronic acid F- 68 (Sigma-Aldrich, USA, cat. no. P1300) at 27°C under stirring.

For the amplification of the plasmids pET-His-GFP and pET-His-PH_(1-110)_, *Escherichia coli* Top10 cells were used, and the expression of recombinant proteins was carried out with *E. coli* BL21(DE3) cells. For the transformation and growth of the bacterial strains, a standard protocol was followed (24). Selection of successfully transformed bacteria was performed on LB (Luria Bertani) agar plates (Sigma-Aldrich, USA, cat. no. L3022-1KG) with kanamycin (50 μg/mL). Bacterial colonies were picked from agar and grown in LB medium (Sigma-Aldrich, USA, cat. no. A7002-250G). Sequencing was performed to assess the presence of the genes of interest.

### Expression and purification of PH_(1-110)_GFP NPs

The process of expression and purification of PH_(1-110)_GFP NPs was previously described (19). Briefly, SF9 cells (2x10^6 cells/ml) were infected with a multiplicity of infection (moi) of 10 with the recombinant baculoviruses. After incubation for 72 hours, cell pellets were obtained by centrifugation. The supernatant was removed, and the pellets were resuspended in phosphate buffered saline (PBS). For the purification process, the cells were sonicated (Qsonica 700, USA). To remove cell debris, the samples were centrifuged, and the supernatant was removed. Finally, PH_(1-110)_GFP NPs were resuspended in PBS.

The total protein concentration was determined with the colorimetric detection of bicinchoninic acid (BCA) from the PierceTM BCA Protein Assay Kit (Thermo Fisher, USA, cat. no. 23225).

### Expression and purification of recombinant proteins GFP and PH_(1-110)_

IPTG (β-D-1-thiogalactopyranoside) was used as an inducer of GFP and PH_(1-110)_ expression as previously described (24) with some modifications. The bacterial solution was cultured at 37°C and 250 rpm until the OD_600_ value reached 0.4–0.8. The induction was carried out with a final concentration of 400 μM of IPTG (Sigma-Aldrich, USA, cat. no. I5502) at 28°C overnight (ON).

Protein purification was performed by affinity chromatography with the Ni-NTA (nickel-nitrilotriacetic acid) Superflow Columns System (Qiagen, Germany, cat. no. 30622). Following the manufacturer’s instructions, GFP was purified under native conditions, while PH_(1-110)_ was purified under denaturing conditions. To administer the treatments to the mice, the proteins were dialyzed with PBS. Finally, the proteins were quantified by the same method described in the previous section.

### Protein electrophoresis and western blot

Purified recombinant proteins PH_(1-110)_GFP NPs, GFP and PH_(1-110)_ were mixed with Laemmli buffer (50 mM Tris-HCL, 3% SDS, 1% β-mercaptoethanol, 20% glycerol, 0.7% bromophenol blue, pH 6.8) and were subjected to 12% sodium dodecyl sulfate - polyacrylamide gel electrophoresis (SDS-PAGE). After electrophoresis, the proteins were stained using Coomassie brilliant blue R-250 (Sigma-Aldrich, USA, cat. No. 112553). Other samples were separated by SDS-PAGE and transferred to nitrocellulose membranes (Merck Millipore, USA, cat. no. HATF00010). The membranes were blocked with 5% fat-free milk in Tris-buffered saline (TBS; 150 mM NaCl; 50 mM Tris-Cl, pH 7.6) ON. Three different primary antibodies were used: anti-GFP (1:1000) (**Figure 1B**), previously produced in our laboratory (19), anti-PH_(1 -110)_ (1:300) (**Figure 1B**) obtained in our laboratory (the process is described later) and anti-PH_(1-110)_GFP (1:300) obtained from a mouse immunized by intranasal route with adjuvant free PH_(1-110)_GFP (**Supplementary Figure 3**). The secondary antibody was horseradish peroxidase-coupled (HRP) anti-mouse IgG (1:5000; Sigma-Aldrich, USA, cat. no. A9044). The membranes were developed using SuperSignal^®^ West Femto substrate (Thermo Fisher, USA, cat. no. 34095), and images were taken with a C-Digit Blot scanner (LI-COR, USA).

### PH_(1-110)_GFP NPs confocal microscopy analysis

Purified PH_(1-110)_GFP NPs were fixed with Fluoromount™ Aqueous Mounting Medium (Sigma-Aldrich, USA, cat. no. F4680). To obtain the images, the FV10i Olympus^®^ confocal microscope with 60 × NA 1.35 oil immersion objective (Olympus^®^, Japan) was used. The excitation and emission filters used were 470 and 520 nm. Images were analyzed using ImageJ software.

### Nanoparticle tracking analysis

NanoSight instrument (Malvern Panalytical, UK) was used to measure the size of PH_(1-110)_GFP NPs. NPs were injected into the sample chamber of the equipment. The NanoSight software (Malvern Panalytical, UK) analyzes the Brownian motion of many particles and by using the Stokes-Einstein equation calculates their diameters (25, 26).

### Determination of the stability of PH_(1-110)_GFP NPs

The stability of fresh PH_(1-110)_GFP NPs and dehydrated PH_(1-110)_GFP NPs stored for two years at room temperature (R.T.D.) was compared. The PH_(1-110)_GFP NPs were dehydrated using the vacufuge™ concentrator 5301 (Eppendorf, Germany) at a centrifugal force of 240 g at 30°C for 30 min and stored at room temperature (28-30°C) for two years. The Agilent Bioanalyzer 2100 (Agilent Technologies, USA) equipped with the Protein 230 assay kit was used to evaluate the degradation of PH_(1-110)_GFP NPs R.T.D. Electrophoretic assays were performed following the manufacturer’s instructions (**Supplementary Figure 2**). Finally, the results were analyzed with Agilent 2100 expert software (Agilent Technologies, USA).

### Fluorescence spectra analysis for GFP and PH_(1-110)_GFP NPs

Aliquots of 500 ng from purified recombinant GFP and PH_(1-110)_GFP NPs were placed on a quartz cuvette and introduced into the analysis chamber of an AMINCO-Bowman series 2 Spectrofluorometer. The excitation wavelength was maintained at 488 nm and the emission was scanned from 300 to 700 nm in a 10 nm step.

### Immunization studies

**Figure 2:**
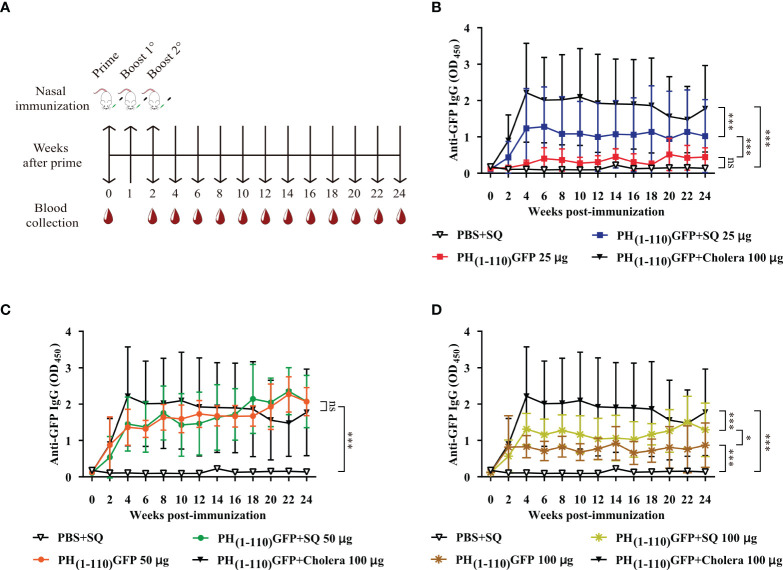
Comparison of IgG antibody response after intranasal administration of PH_(1-110)_GFP NPs at different concentrations with and without adjuvant. **(A)** Schematic timeline of immunizations and bleeds of BALB/c mice. The immunization schedule included three doses and bleeds at 2-week intervals. **(B–D)** Measurement of anti-GFP antibodies by ELISA; PH_(1-110)_GFP 25 μg with and without adjuvant **(B)**, PH_(1-110)_GFP 50 μg with and without adjuvant **(C)**, and PH_(1-110)_GFP 100 μg with and without adjuvant **(D)**. Control groups: PBS + SQ and PH_(1-110)_GFP + cholera toxin 100 μg. Adjuvants: squalene (SQ) and cholera toxin (6 μg). Data represent mean ± SD (n = 4). The *p* values were determined by Two-way ANOVA with Tukey post-tests. *p < 0.033; ***p < 0.001; ns = not significant.

6-8-week-old female BALB/c mice were purchased for all experiments. The administration of the different treatments was performed by intranasal route (IN). The inoculum was prepared in 12 μl final volume and divided over both nostrils using a pipette. All mice received 3 doses of the treatment on days 0, 14, and 21 (**Figure 2A**). The adjuvants used were Squalene (SQ; OZ Biosciencies, France, cat. no. 34095) in 1:1 dilution and Cholera toxin (6 µg) donated by Dr. Juan Carlos Gomora (Institute of Cellular Physiology, UNAM). Blood samples were collected on day 0 and at two-week intervals until the end of the study. Blood samples were centrifuged, and the sera were stored at −70°C until analysis by Enzyme-Linked Immunosorbent Assay (ELISA). The animals were euthanized in a CO2 chamber following the CICUAL guidelines and Mexican Official Standard NOM-062-ZOO-1999. Characteristics of some experiments are described in the corresponding section.

### Dose-response studies

Eight groups of mice were immunized (n = 4) intranasally following the scheme described in **Figure 2A**. The treatments were: 1) PBS+SQ (12 µl, negative control); 2) PH_(1-110)_GFP 25 µg; 3) PH_(1-110)_GFP+SQ 25 µg; 4) PH_(1-110)_GFP 50 µg; 5) PH_(1-110)_GFP+SQ 50 µg; 6) PH_(1-110)_GFP 100 µg; 7) PH_(1-110)_GFP+SQ 100 µg and 8) PH_(1-110)_GFP+Cholera 100 µg (positive control). Blood samples were taken at 2-week intervals for 24 weeks. From the results obtained, the dose of 50 µg was chosen for the subsequent experiments.

### Measuring long-lasting anti-GFP antibodies

The mice were immunized *via* IN (**Figure 2A**) with the following treatments (n = 4): 1) PBS+SQ (12 µl, negative control); 2) GFP 50 µg; 3) GFP+SQ 50 µg; 4) PH_(1-110)_GFP 50 µg; 5) PH_(1-110)_GFP+SQ 50 µg and 6) PH_(1-110)_GFP+Cholera 100 µg (positive control). Blood samples were taken at 2-week intervals for 24 weeks. The collected sera served to compare the duration of the specific anti-GFP antibodies and to detrmine the advantage of administering PH_(1-110)_GFP nasally without the use of an adjuvant. In addition, specific anti-GFP antibody titers were determined at weeks 4, 14, and 24 (**Figure 4A**). To assess whether the nanocarrier (PH_(1-110)_) generated a response against itself, specific anti-PH_(1-110)_ antibody titers were measured at weeks 4, 14, and 24 (**Figure 4B**). In addition, different concentrations of PH_(1-110)_ were used in the ELISA assay to ensure the disposition of PH_(1-110)_ in the wells and not hide the presence of anti-PH_(1-110)_ antibodies (**Supplementary Figure 4A**). Sera from mice immunized with: 1) PBS+Alum IM (50 µl), from a previously published experiment (19) and 2) GFP IN 50 µg, were used as negative controls. The test sera were: 3) PH_(1-110)_GFP IM 25 µg (previously published experiment) (19); 4) PH_(1-110)_GFP IN 50 and 5) PH_(1-110)_GFP+Alum IM 25 µg. To generate a positive control, mice (N = 4) were immunized subcutaneously (SC) with the recombinant protein PH_(1-110)_ with two doses of 25 µg with Aluminum Hydroxide (Alum) as an adjuvant.

### Immunological memory evaluation

Immunological memory was assessed at week 24 of the *Specific Long-Lasting anti-GFP Antibodies* experiment (**Figure 5A** red box). 10 µg of GFP without adjuvant was administered in all groups to restimulate the production of anti-GFP antibodies in case of having previously mounted a response. Blood samples were obtained at 0-, 1-, 2-, and 3-weeks post-challenge. Anti-GFP antibody levels were measured by ELISA.

### Th1/Th2 humoral immune response

At week 4 post-immunization, the IgG1, IgG2a, and IgG2b isotypes of immunoglobulins were measured by ELISA (**Figure 6**). IgG2a and IgG2b are Th1-related isotypes and IgG1 is Th2-related isotype. To determine the predominance of Th1 or Th2 response, the IgG2a/IgG1 ratio was determined (27, 28).

### Identification of IgM and IgA in serum and BALF

**Figure 3:**
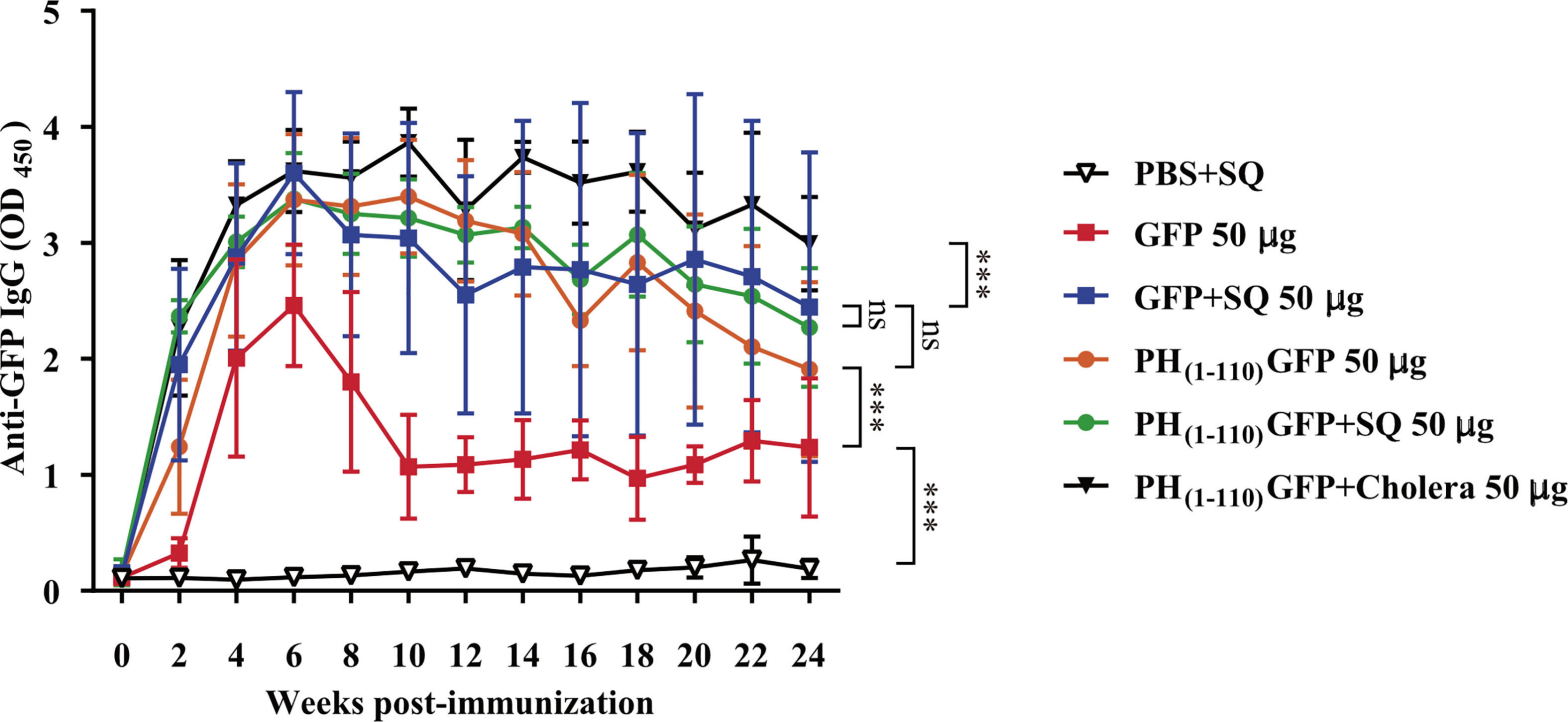
Induction of long-lasting systemic immune response by PH_(1-110)_GFP NPs administered nasally. Comparison of the duration of systemic specific antibodies against GFP. Antibodies were detected by ELISA assay. Treatments included free GFP and PH_(1-110)_GFP NPs, both treatments were administered with and without adjuvant. The control groups were: PBS + SQ and PH_(1-110)_GFP + cholera toxin. Adjuvants: squalene (SQ) and cholera toxin (6 μg). Data represent mean ± SD (n = 4). The *p* values were determined by Two-way ANOVA with Tukey post-tests. ***p < 0.001; ns = not significant. The timeline of immunizations and bleeds is the same as in **Figure 2A**.

New groups of mice were immunized *via* IN with the treatments described in **Figure 3** (n = 6-7): 1) PBS+SQ (12 µl, negative control); 2) GFP 50 µg; 3) GFP+SQ 50 µg; 4) PH_(1-110)_GFP 50 µg; 5) PH_(1-110)_GFP+SQ 50 µg and 6) PH_(1-110)_GFP+Cholera 100 µg (positive control). Serum IgM and IgA immunoglobulins were measured; IgM was measured at week one post-immunization because it is an early-onset immunoglobulin (29), and IgA was measured at week 3 post-immunization.

Following the previously described protocol (30), Bronchoalveolar Lavage Fluid (BALF) was collected. Briefly, mice were euthanized 1 week after the last immunization. An incision was made in the chest at the level of the trachea. After exposing the trachea, a catheter was inserted and 500 μl of saline with 100 μM ethylenediaminetetraacetic acid (EDTA) was administered. The content was gently aspirated and placed in a tube. To obtain more BALF, the saline administration was repeated. The samples obtained were centrifuged and stored at -70°C until use. Finally, IgM and IgA were measured in the BALF by ELISA.

### Detection of anti-GFP and anti-PH_(1-110)_ antibodies

Anti-GFP or anti-PH_(1-110)_ antibodies were measured depending on the experiment. The ELISA microplates (Corning, USA, cat. no. 3590) were coated with 50 µl of GFP (1 µg/ml) or PH_(1-110)_ (0.5, 1, 2, 4, 8, 16 or 32 µg/ml) in 0.1 M sodium bicarbonate buffer (pH 9.2) ON at 4°C. The microplates were washed and subsequently blocked with PBS-Triton + 5% fat-free milk for 1 h at 37°C. Then, 50 µl of the sera (1:100) or BALF (1:1) were added to each well. For the evaluation of immunologica memory, the sera were diluted 1:1600. For the antibody titration, 2-fold serial dilutions of the sera were performed (1:100 to 1:12,800). The plates were incubated for 1 h at 37°C or 4°C. After washing the plates, 50 µl of the secondary antibodies were added: anti-mouse IgA (1:3000; Thermo Fisher, USA, cat. no. 626720); anti-mouse IgG (1:5000; Sigma-Aldrich, USA, cat. no. A9044); anti-mouse IgG1 (1:3000; Thermo Fisher, USA, cat. no. 04-6120); anti-mouse IgG2a (1:3000; Abcam, UK, cat. no. ab98698); anti-mouse IgG2b (1:3000; Thermo Fisher, USA, cat. no. 610320) or anti-IgM (1:2000; Invitrogen, USA, cat. no. 61032062-6820), all HRP-conjugated, and the plates were incubated 1 h at 37°C. After the last wash, 50 µl/well of the substrate 3,3’,5,5’-Tetramethylbenzidine (TMB) (Sigma-Aldrich, USA, cat. no. 00-2023) were added, and the microplates were incubated at RT for 20 min. To stop the reaction, 50 µl/well of 0.16 M Sulfuric acid solution was added. The results were measured using a multiskan FC 3.1 microplate reader (Thermo Fisher, USA).

### Proliferation assay

Five groups of mice were immunized with the following immunogens (N = 3): 1) PBS+SQ (12 µl, negative control); 2) PH_(1-110)_GFP IM 25 µg; 3) PH_(1-110)_GFP+FA IM 25 µg; 4) PH_(1-110)_GFP IN 50 µg; 5) PH_(1-110)_GFP+SQ IN 50 µg. The treatment of mice from groups 2 and 3 was carried out following the protocol previously described (19). Mice were euthanized at week 3 post-immunization, and the procedure to isolate splenocytes was started. The spleen of each mouse was removed and perfused with RPMI 1640 medium (Thermo Fisher, USA, cat. no. 31800022) to isolate splenocytes. Lysis buffer (NH_4_Cl 155 mM; KHCO3 10 mM; EDTA.2Na 97.7 μM) was added to the cells to remove erythrocytes, and then splenocytes were incubated with 5-(and-6)-Carboxyfluorescein Diacetate, Succinimidyl Ester (CFSE) at a concentration of 5 μM (Thermo Fisher, USA, cat. no. C1157). Cells were plated on culture plates for stimulating with 1) Concanavalin A (ConA; 3 µg/ml) (Sigma-Aldrich, USA; cat. no. C-5275), Bovine Serum Albumin (BSA; 20 µg/ml); 2) PH_(1-110)_GFP (20 µg/ml) or 3) GFP (20 µg/ml) and were incubated for 5 days at 37°C in a 5% CO2 humidified atmosphere.

Cell proliferation was evaluated 5 days after starting splenocyte stimulation. Harvested cells were labeled with Zombie Aqua™ (BioLegend^®^, USA, cat. no. 423102) to determine viability and with Phyocoerythrin Cyanin 5.1 (PE-Cy™5)-conjugated anti-CD3 antibody (BD ​​Biosciences, USA, cat. no. 553065) to identify the population of T cells. The proliferation index was determined by measuring CFSE fluorescence. The result was expressed as the number of divisions/the number of divided cells. At least 10,000 events were collected to analyze proliferation by flow cytometry using the Attune NxT Acoustic Focusing Cytometer (blue/red/violet/yellow laser) (Thermo Fisher, USA). A summary of the strategy to capture the data is shown in **Supplementary Figure 4A**. The final analysis was performed using FlowJo 10.8.0 software (FlowJo LLC, USA)

### Statistical analysis

GraphPad Prism 7 software (GraphPad software, USA) was used for the statistical analysis of each experiment. The results express the mean ± SD. The tests used for multiple comparisons were two-way ANOVA with Tukey or Dunnett post-tests. The cut-off was determined as the mean + 2 standard deviations. All experiments were repeated at least once. *p < 0.033; **p < 0.002; ***p < 0.001; ns = not significant.

## Results

### Production, purification and characterization of PH_(1-110)_GFP NPs

The cDNA encoding the first 110 amino acids from Autographa californica polyhedrin (31) was cloned in-frame at the 5´of the cDNA encoding the enhanced green fluorescent protein (GFP, V35620 Invitrogen) to produce the PH_(1-110)_GFP gene. PH_(1-110)_GFP was cloned in the pFastbac transfer vector under the polyhedrin promoter to generate the recombinant baculovirus expressing the PH_(1-110)_GFP fusion protein (**Figure 1A**). GFP was used as a model antigen for two main reasons: 1) the fluorescent protein allows the rapid characterization of the nanoparticles produced by PH_(1-110)_GFP and, 2) GFP is poorly immunogenic and thus any enhanced antigenicity conferred by PH_(1-110)_ would be easily identified. Furthermore, the fluorescence of GFP depends heavily on its tertiary protein structure intact (32, 33). Since maintaining the native tertiary protein structure of an antigen is necessary to obtain an adequate immune response that recognizes the original pathogen, the use of GFP allow us to explore the tertiary protein structure of GFP indirectly by analyzing its emission spectra and comparing it with the spectra obtained with free GFP (not fused to PH_(1-110)_). Indeed emission spectra from free GFP and that obtained with PH_(1-110)_GFP NPs was indistinguishable (**Supplementary Figure 1A**). Furthermore, the emission spectra from newly produced PH_(1-110)_GFP is indistinguishable from that of PH_(1-110)_GFP NPs stored for 2 years (**Supplementary Figure 1B**).

Low-speed centrifugation (Materials and Methods) results in the fast and efficient purification of PH_(1-110)_GFP NPs (**Figure 1B**). A single protein band in the polyacrylamide gel is observed (**Figure 1B** left panel). Purity of PH_(1-110)_GFP recombinant protein was comparable with that obtained from recombinant GFP purified by affinity chromatography (**Figure 1B** left panel). Western blot analysis identified single bands for GFP and PH_(1-110)_GFP using both anti-GFP and anti- PH_(1-110)_ antibodies (**Figure 1B** right panel).

Purified PH_(1-110)_GFP NPs are clearly visible under confocal microscopy showing particles of regular sizes (**Figure 1C**). Further sizing analysis of the purified PH_(1-110)_GFP NPs using a nanoparticle analyzer (Material and Methods), showed particles of different sizes ranging from 100-400 nm (**Figure 1D**). Although PH_(1-110)_GFP NPs appear polydisperse at first sight, the great majority of nanoparticles (over 70%) displayed sizes smaller than 200 nm (**Figure 1D**).

To evaluate the thermostability of the PH_(1-110)_GFP NPs we analyzed freshly produced nanoparticles using an electrophoretic method (Material and Methods) and compared the results obtained with those produced using PH_(1-110)_GFP NPs stored for 2 years at room temperature dehydrated (R.T.D.). The electrophoretic analysis indicate that no protein degradation occurred during the 2 year R.T.D., since the fresly produced PH_(1-110)_GFP accounted for 91% of the total sample while with the 2-year stored PH_(1-110)_GFP the number was over 89% (**Supplementary Figure 2**). All these results indicate that PH_(1-110)_ prevents antigen degradation when stored at room temperature dehydrated for long periods of time, conferring thermostability to the carried antigen.

Therefore, with the baculovirus-insect cell expression system large amounts of PH_(1-110)_GFP NPs can be produced rather rapidly. This procedure can expedite the response times to emerging diseases in terms of vaccine generation. Another relevant aspect is the ease of purification by using a simple centrifuge, yielding high purity nanoparticles with sufficient purity for nasal vaccination.

### Nasal immunization with PH_(1-110)_GFP NPs results in the production of antibodies that remained elevated for months

A nasal immunization protocol was established using mice to explore the immunogenicity of the pure PH_(1-110)_GFP NPs. The protocol consisted in 3 nasal immunizations 1 week apart, with three different protein concentrations of the nanoparticles: 25, 50 and 100 ug in 6 uL per nostril (Material and Methods). Blood samples were drawn every week for 24 weeks to assess circulating antibodies anti-GFP (**Figure 2A**). Most interestingly, circulating IgG antibodies anti-GFP were identified in the serum of immunized animals 2 weeks after immunization (**Figures 2B–D**). All three PH_(1-110)_GFP NPs generated anti-GFP antibodies (**Figures 2B–D**). Application of PH_(1-110)_GFP NPs in combination with 6 ug of the B subunit from cholera toxin or 1:1 squalene dilution (two robust adjuvants for nasal immunization) did not improve antibody generation (**Figures 2B–D**). This result indicate that PH_(1-110)_GFP NPs are sufficient to produce a robust immune response and that adjuvants do not improve the immunogenicity of the nanoparticles. Contrary to this observation, GFP without adjuvants produced a small transient response with antibody production decaying after week 6 post-immunization (**Figure 3**). After 24 weeks antibodies induced with PH_(1-110)_GFP NPs remained elevated; in contrast, those induced with GFP reached the baseline. Combining GFP with squalene (SQ) resulted in antibody levels equivalent to those obtained with PH_(1-110)_GFP NPs without adjuvant (**Figure 3**).

### Nasal immunization with PH_(1-110)_GFP NPs results in high titer antibodies against GFP but not PH_(1-110)_

**Figure 4:**
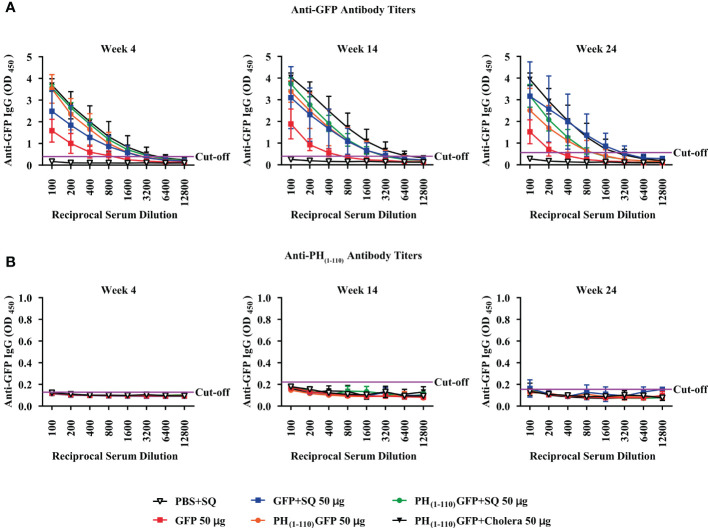
Production of high titers of specific antibodies against the GFP antigen and not against the nano-carrier PH_(1-110)_. Serial 2-fold dilution of sera from immunized mice to determine the titers of specific antibodies against GFP **(A)** and against PH_(1-110)_ **(B)**; for both the titers were determined at week 4, 14, and 24. The horizontal purple line shows the cut-off point. Data represent mean ± SD (n = 4).

Antibody titers were measured for all the conditions explored with and without adjuvant with GFP and PH_(1-110)_GFP NPs. As illustrated in **Figure 4**, high antibody titers remained present in animals immunized with PH_(1-110)_GFP NPs and GFP combined with squalene for 24 weeks (**Figure 4A**). Even though we did not explore time points beyond 24 weeks, comparing the titers obtained at weeks 14 and 24 clearly show that antibodies remained elevated with no significant changes, suggesting that antibodies may remained elevated far longer than 24 weeks.

Most surprisingly, we could not detect antibodies against PH_(1-110)_ at any of the time points explored, indicating that PH_(1-110)_ is poorly immunogenic (**Figure 4B**). To demonstrate that PH_(1-110)_ was present in the ELISA plate, we conducted a control experiment using a previously generated antibody against PH_(1-110)_ produced in bacteria system (**Supplementary Figure 3A**) and, we also demonstrate that there is no presence of linear antibodies anti-PH_(1-110)_ by Western Blot (**Supplementary Figure 3B**). This results shows that the antigen (PH_(1-110)_) is present but the serum from immunized animals does not contain measurable antibodies against PH_(1-110)_.

### Nasal immunization with PH_(1-110)_GFP NPs generates immunological memory

**Figure 5:**
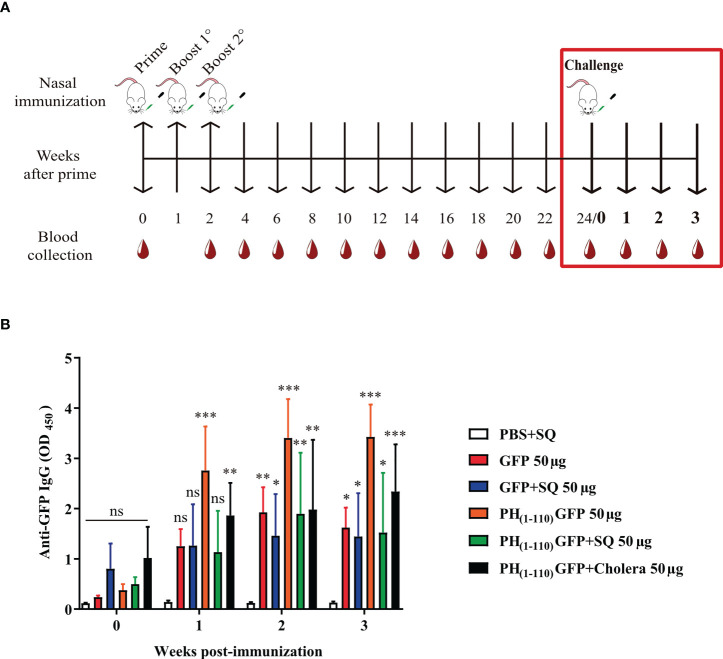
Generation of antibody memory response by nasal immunization with PH_(1-110)_GFP NPs. **(A)** Schematic timeline of the memory test (red box). The initial immunization schedule is described in **Figure 2A**. At week 24 post-immunization, a single dose of 10 µg GFP without adjuvant was administered intranasally (day 0 for the challenge) to each group. **(B)** Measurement of the increase in antibodies at week 1, 2, and 3 post-challenge. Specific IgG antibodies against GFP were measured by ELISA assay. Data represent mean ± SD (n = 4). All groups were compared against the PBS+SQ control group. The *p* values were determined by Two-way ANOVA with Dunette post-tests. *p < 0.033; **p < 0.002; ***p < 0.001; ns = not significant.

To evaluate the possibility that nasal immunization with PH_(1-110)_GFP NPs may generate long lasting immunological memory we challenged previously immunized animals with the different antigens explored (**Figure 5A**). After 24 weeks of the initial immunization, animals were challenged again with the antigen GFP (10μg). We observed a response after 1 week of the challenge only in animals previously immunized with PH_(1-110)_GFP NPs but without adjuvant and when cholera was added to the NPs; in contrast, animals previously immunized with GFP, GFP+SQ or PH_(1-110)_GFP+SQ did not respond (**Figure 5B**). 3 weeks after the challenge we observed a small increment in antibodies from animals previously immunized with GFP+SQ (**Figure 5B**). The response obtained using PH_(1-110)_GFP NPs with and without cholera toxin was identical, indicating that immunological memory is not improved by the use of an adjuvant with our nanoparticles (**Figure 5B**).

### Evaluation of the Th1/Th2 humoral immune response

**Figure 6:**
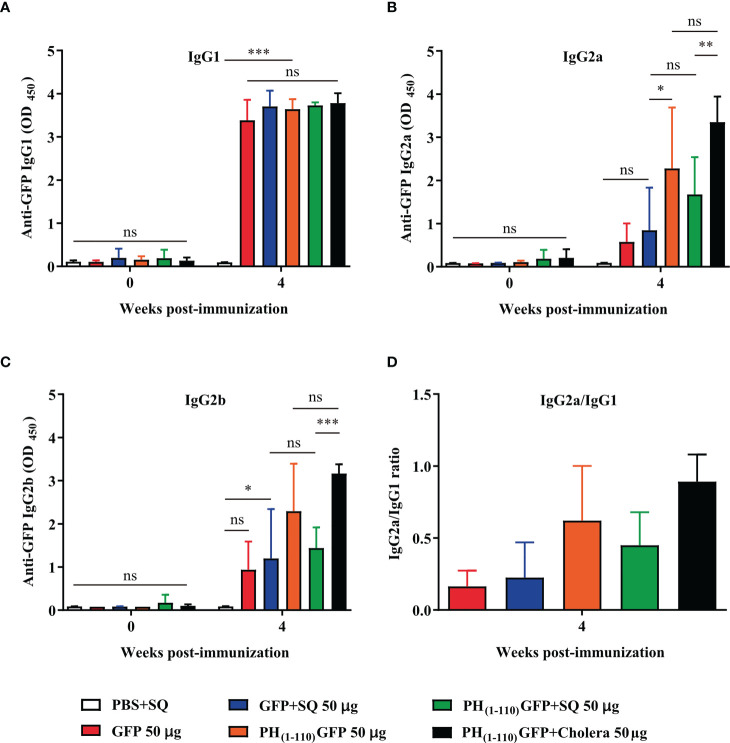
Nasal immunization with PH_(1-110)_GFP NPs favors the Th2 response. Two weeks (week 4) after the last administration of the PH_(1-110)_GFP NPs and GFP treatments, the profile of immunoglobulin G subtypes was evaluated; IgG1 **(A)**, IgG2a **(B)**, and IgG2b **(C)**. IgG isotypes were measured by ELISA assay. **(D)** The IgG2a/IgG1 ratio was evaluated in each group to determine the presence of Th1, Th2 or both responses. Data represent mean ± SD (n = 4). The *p* values were determined by Two-way ANOVA with Tukey post-tests. *p < 0.033; **p < 0.002; ***p < 0.001; ns = not significant.

We evaluated the type of immunoglobulins present in the sera from immunized animals. We observed IgG1, IgG2a and IgG2b in sera from animals immunized with PH_(1-110)_GFP NPs (**Figures 6A–C**). However, the IgG2a/IgG1 ratio suggests an enhanced Th2 immune response (**Figure 6D**). IgG1 was obtained with the immunization using GFP+SQ, but IgG2a could not be detected and the presence of IgG2b was minimal (**Figure 6B**). This result clearly support the fact that the type of immunoglobulins produced by the immunization with PH_(1-110)_GFP NPs is different from that obtained with the immunization using GFP+SQ as antigen.

We conducted experiments directed to evaluate T cell proliferation in animals immunized with the different antigens and conditions were explained above. However, we did not observe any T cell proliferation in cells obtained from animals immunized with PH_(1-110)_GFP NPs or with GFP+SQ, even though cells proliferated normally when using the positive control Concanavalin A (**Supplementary Figure 4**). These results indicate that nasal immunization with PH_(1-110)_GFP NPs provokes a strong humoral response but no cellular immunity appear to participate. Thus, antibodies produced by B cells appear to be the main mechanism responsible for the immune response observed after nasal immunization with PH_(1-110)_GFP NPs.

### Nasal immunization results in elevated antibodies in bronchoalveolar lavage fluid (BALF)

**Figure 7:**
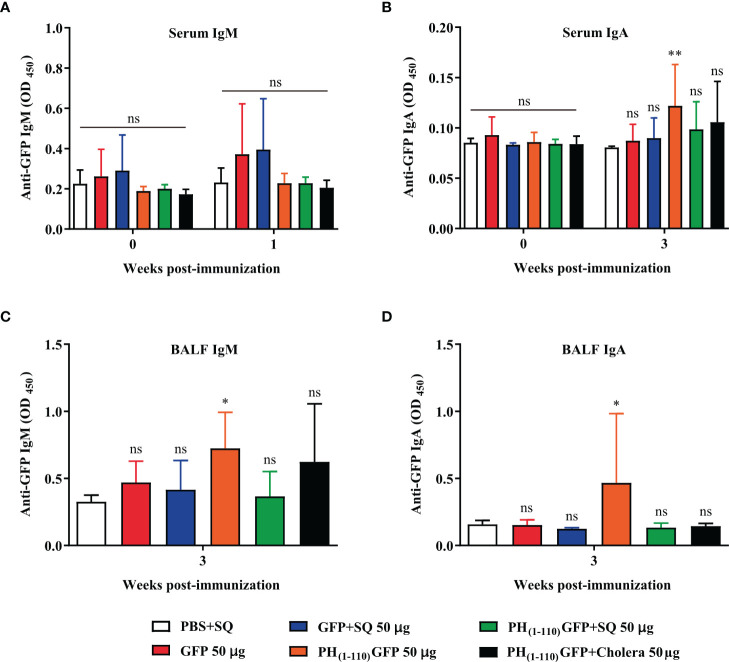
The presence of IgM and IgA in BALF and serum demonstrate the induction of systemic and local responses by PH_(1-110)_GFP NPs. **(A)** Evaluation of the presence of IgM in serum. IgM was evaluated in the first week post-immunization because it is an immunoglobulin that is produced in the first days of the immune response. **(B)** The presence of serum IgA was assessed at week 3 post-immunization when response to all 3 doses of treatments was established. The procedure to obtain Bronchoalveolar Lavage Fluid (BALF) from mice requires euthanasia, for this reason only the presence of IgM **(C)** and IgA **(D)** was evaluated at week 3 post-immunization. The determination of IgM and IgA immunoglobulins was performed by ELISA assay. Data represent the mean ± SD (For PH_(1-110)_GFP +cholera, n = 6; for the other groups, n = 7). All groups were compared against the PBS+SQ group. The *p* values ​​were determined by Two-way ANOVA with Dunette post-tests. *p < 0.033; **p < 0.002; ns = not significant.

Given the results obtained thus far indicating that the nasal immunization with PH_(1-110)_GFP NPs induce a humoral immune response, we conducted experiments to evaluate other immunoglobulins present in the sera and in bronchoalveolar lavage fluid (BALF), to evaluate the mucosal immune response. We did not observe IgM immunoglobulin in the sera of any of the animals immunized with the different antigens and conditions explored (**Figure 7A**). We observed IgA in the sera from animals immunized with PH_(1-110)_GFP NPs (**Figure 7B**). Most interestingly, no IgA was observed in the sera from animals immunized with PH_(1-110)_GFP+SQ and PH_(1-110)_GFP+cholera (**Figure 7B**). However, IgG2b was clearly detected (**Figure 7C**). Furthermore, we detected IgM and IgA in BALF from animals immunized with PH_(1-110)_GFP NPs only. Combining the PH_(1-110)_GFP NPs with adjuvants (squalene or cholera toxin) prevented the generation of IgM and IgA, strongly suggesting that the use of these adjuvants altered the mucosal immune response to PH_(1-110)_GFP NPs. The ratio IgG2a/IgG1 suggested a combined TH1-TH2 immune response (**Figure 7D**).

## Discussion

In the present study we have shown that using the first 110 amino acids from the occlusion body protein, polyhedrin it is possible to generate nanoparticles that may carry antigens. In combination with the baculovirus-insect cell expression system, it is possible to produce NPs carrying the antigen of interest, which can be purified with a single low-speed centrifugation step.

Previously we had shown that the administration of GFP intramuscularly without adjuvant did not generate an antibody response, but only when coupled to our nanoparticles we can detect selective antibodies against the antigen (19). However, nasal immunization has not been explored with PH_(1-110)_GFP NPs until the present study.

It has been shown that intranasal vaccination can improve the local response against respiratory pathogens (34, 35). The immune response we obtained with nasal immunization is characterized by the production of IgG and IgA immunoglobulins in serum and IgM and IgA in mucosa (measured in bronchoalveolar lavage fluid).

Most strikingly, the addition of adjuvants (squalene or the B subunit from cholera toxin) altered the type of immunoglobulins produced by the nasal immunization with PH_(1-110)_GFP NPs, indicating that adjuvants affect the immune response induced by PH_(1-110)_GFP NPs. It is a well-known phenomenon that adjuvants alter the immune response (36). Adjuvants can alter the quality and quantity of adaptive immune responses (36).

Although several studies have shown the induction of both humoral and cellular immune responses by intranasal vaccination (37, 38), nasal immunization with PH_(1-110)_GFP NPs did not induce T cell proliferation, indicating that the cellular immune system may not participate in the response to this antigen. On the other hand, it is known that there is a T-independent response (little or no participation of T lymphocytes) mainly against antigens made up of polysaccharides, but some protein antigens of viral origin have been shown to generate a T-independent responses (39, 40). In addition, this type of antigen generates memory of B cells (40, 41) as in the case of our study (**Figure 5B**). However, more studies are needed to further confirm this observation, which may include measuring cytokines produced and the type of lymphocytes involved in the immune response.

Polyhedrin main function is the production of occlusion bodies, in fact polyhedral is formed mainly by polyhedrin. The natural role of polyhedra is to maintain baculoviruses viable for many years, resisting harsh environmental conditions such as fluctuating temperatures, changes in humidity, pH, prolong exposure to UV light and many others.

Studies conducted by our group indicate that many of the preservative functions found in polyhedrin are also present in PH_(1-110)_. Most interestingly, GFP fluoresces even when incorporated into the nanoparticles (**Figure 1C**), indicating that the tridimensional structure of GFP (which is required for its fluorescent functionality) (32, 33, 42, 43), is preserved inside the PH_(1-110)_GFP NPs. We have maintained PH_(1-110)_GFP NPs for several years at room temperature dehydrated and the fluorescence of GFP remains unaltered (**Supplementary Figure 1**). Furthermore the emission spectra of free GFP is indistinguishable from that of PH_(1-110)_GFP NPs (**Supplementary Figure 1A**). Strikingly, the emission spectra of freshly produced PH_(1-110)_GFP is inseparable from that obtained with PH_(1-110)_GFP stored for 2 years (**Supplementary Figure 1B**). No signs of protein degradation were observed in electrophoretic analysis of PH_(1-110)_GFP samples stored for 2 years at room temperature (**Supplementary Figure 2**).

Intranasal immunizations are simple, easy, convenient, and safer than other routes of vaccination. Over the last few years there have been several attempts to develop adequate carriers for vaccines to be used in nasal immunizations (44–49). Several formulations have been explored such as dry powder, gels, aerosols and drops among others (50). However, mucosal delivery of antigens remains a complex challenge (51). The use of nanoparticles based on PH_(1-110)_ provides several advantages: First the PH_(1-110)_ sequence is poorly immunogenic, as demonstrated in the present study, second PH_(1-110)_ NPs are easy and inexpensive to produce and purify. Vaccine purity of 90% or less may not be sufficient for intramuscular or subdermal vaccines, but for nasal immunization may be adequate. Third and not less important, thermostable nanoparticles may remain longer in the respiratory tract, increasing the possibilities for the immune system to react and produce a robust immune response.

One of the first barriers at the nasal cavity against foreign bodies is hair at the entrance to the nares, the nostrils, which successfully keeps out larger particles. Therefore, the use of smaller particles may facilitate antigen delivery. In this regard, the PH_(1-110)_GFP NPs used in the present study ranged in sizes from 100-200 nm, with a smaller proportion of nanoparticles in the 300-400 nm range (**Figure 1D**). The surface of the nasal cavity is covered with a mucus layer, which traps smaller particles and prevents their travel into deeper areas of the respiratory tract. Mucociliary clearance oversees removal of trapped particles moving them out of the respiratory system. The mucosal immune system provides local protection against pathogens. The mucosal immune system then produces the immunoglobulin A (IgA). Most nasal vaccines explored elevate IgA. Rather surprising finding from our study was the presence of systemic IgG in the blood stream as a response of nasal immunization with PH_(1-110)_GFP NPs. Also surprising was the finding of anti-GFP IgA in the sera of immunized animals, since only small amounts of IgA can be found in the serum with the majority of IgA found in external secretions, also known as secretory IgA (sIgA).

Despite all the obvious advantages of using nasal vaccines, the use of particulate nasal vaccines may not come without some drawbacks. Highly stable particles that may remain in the respiratory tract for weeks or even months may have undesirable side effects. One of the most obvious drawbacks that must be explored is inflammation. Using a nanoparticle-based platform for the generation of multiple vaccines may imply the constant presence of such particles in the respiratory tract. If the particles induced chronic inflammation, this may result in other unforeseen problems that need to be explored. Therefore, an adequate evaluation of the toxicity of prolonged exposure to protein-based nanoparticles should be evaluated in detail.

Unfortunately, there are not commercially available nasal vaccines to this date. One of the most successful public nasal vaccines was Flumist, a well-tolerated, good efficacy nasal spray from MedImmune (MD, USA) directed against influenza. Flumist was discontinued in 2017 due to some side effects including runny nose/nasal congestion (52).

Exploring new antigen carriers for nasal vaccination is the first step towards the successful design of nasal vaccines. The results presented here are encouraging towards the use of PH_(1-110)_ NPs as antigen carriers, although further studies using antigens from pathogens are required to validate this platform. Toxicity studies and the evaluation of side effects are also needed. We are currently working on the PH_(1-110)_ platform to develop nanoparticles that carry several fragments from the Spike glycoprotein from SARS-CoV-2, including the entire Receptor Binding Domain (RBD). Although the preliminary results are encouraging, ongoing protocols will determine if any of the nanoparticles may function as a safe and efficient nasal vaccine.

## Data availability statement

The raw data supporting the conclusions of this article will be made available by the authors, without undue reservation.

## Ethics statement

The animal study was reviewed and approved by Comite de etica. Instituto de Fisiologia Celular. UNAM.

## Author contributions

AC-R, GA, AS, CS, MJR-P and LV conducted experiments and analyzed data. GG, LE and MM analyzed data and contribute to the manuscript writing. GG, LE and LV wrote the initial version of the manuscript. All authors revised and approved the final version. All authors contributed to the article and approved the submitted version.

## Funding

This work was supported by grant AV200320 from Dirección General de Asuntos del Personal Académico (DGAPA) to LV and a grant from Consejo Nacional de Ciencia y Tecnología (Conacyt) A-S8731 to LE.

## Acknowledgments

The present study was approved by protocol HIM 2020/022 from Hospital Infantil de México Federico Gómez granted to MM. We would like to thank the technical assistance from Aarón Dalí López Lorea and César Jusif Cabadas Norberto, Facultad de Ciencias, UNAM. We would also like to thank the Unidad de Biología Molecular, Unidad de Cómputo, Bioterio y al Taller de Mantenimiento electrónico, eléctrico y mecánico, all these services of the Institute of Cellular Physiology, UNAM.

## Conflict of interest

The authors declare that the research was conducted in the absence of any commercial or financial relationships that could be construed as a potential conflict of interest.

## Publisher’s note

All claims expressed in this article are solely those of the authors and do not necessarily represent those of their affiliated organizations, or those of the publisher, the editors and the reviewers. Any product that may be evaluated in this article, or claim that may be made by its manufacturer, is not guaranteed or endorsed by the publisher.
