# Supplementary material for: An ambient-temperature stable nanoparticle-based vaccine for nasal application that confers long-lasting immunogenicity to carried antigens
Source: Front Immunol. 2022 Oct 31;13:1057499. doi: 10.3389/fimmu.2022.1057499 (PMC9660247; doi:10.3389/fimmu.2022.1057499)
Supplement: Supplementary file 1 [file DataSheet_1.docx]

Supplementary Material

## Supplementary Figures

**Supplementary Figure 1.** Stability of GFP inside the PH_(1-110)_GFP nanoparticles. **(A)** comparison of the emission spectra for GFP (red) and PH_(1-110)_GFP (blue). **(B)** comparison of the emission spectra for freshly produced PH_(1-110)_GFP and PH_(1-110)_GFP (fuchsia) after 2 years of storage at room temperature (green). Notice that all emission spectra are indistinguishable from each other, indicating that the fluorescence properties of GFP are unaltered. In all conditions the excitation was 395 nm (Material and Methods).

**Supplementary Figure 2.** Dehydrated PH_(1-110)_GFP nanoparticles remain stable after two years at room temperature. Bioanalyzer microelectrophoresis **(A)** and Bioanalyzer electropherogram **(B)** of freshly purified PH_(1-110)_GFP nanoparticles. Bioanalyzer microelectrophoresis **(C)** and Bioanalyzer electropherogram **(D)** of dehydrated PH_(1-110)_GFP nanoparticles kept at room temperature for two years. For analysis in the Bioanalyzer, the nanoparticles were resuspended in PBS. The molecular weight of the PH_(1-110)_GFP protein is ~42 kDa.

**Supplementary Figure 3.** The PH_(1-110)_ nanocarrier does not generate specific antibodies against itself. **(A)** Comparison of the presence of specific antibodies against PH_(1-110)_ by the administration of different treatments and routes of administration of PH_(1-110)_GFP. Negative control sera: PBS+Alum and GFP IN; as positive control PH_(1-110)_+Alum SC. Adjuvant: Alum (Alumnium Hydroxide). IM: intramuscular, IN: intranasal and SC: subcutaneous. **(B)** Western blot assay to assess the presence of linear epitope antibodies against PH_(1-110)_ after nasal administration of PH_(1-110)_GFP without adjuvant. Lane M: molecular mass standards; Lane 1: SP(1-110); Lane 2: PH_(1-110)_GFP; Lane 3: GFP. Blue arrow: PH_(1-110)_GFP (~42 kDa); Green arrow: GFP (~28 kDa); Gray arrow: PH_(1-110)_ (~19 kDa). Data represent mean ± SD (n = 4). The *p* values were determined by Two-way ANOVA with Tukey post-tests. **p* < 0.033; ***p < 0.001; ns = not significant.

**
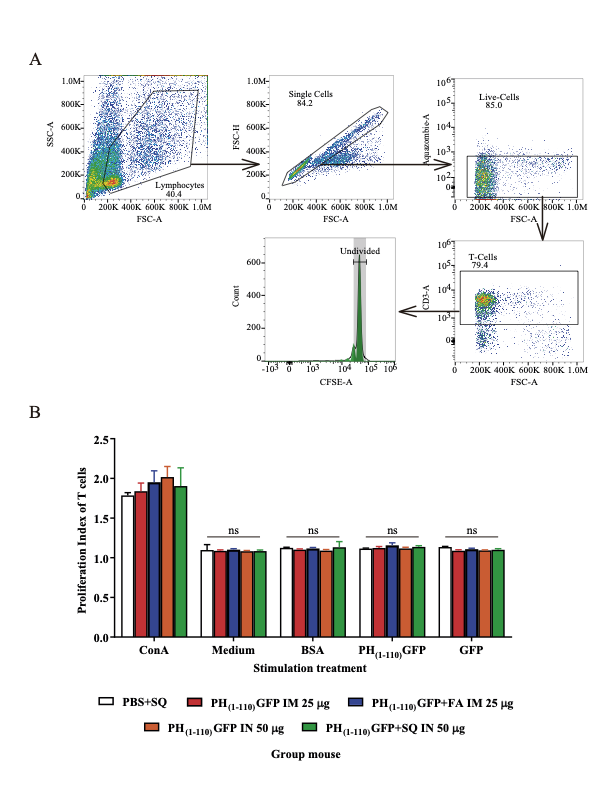
**

**Supplementary Figure 4.** Nasal immunization with PH_(1-110)_GFP nanoparticles does not stimulate T-lymphocyte proliferation. **(A)** General strategy for data acquisition in the cytometry system. The identification of T lymphocytes was done with anti-CD3. To assess proliferation, T lymphocytes were stained with CFSE. **(B)** Determination of the proliferation index of T lymphocytes. PH_(1-110)_GFP nanoparticles were administered intramuscularly (IM) and intranasally (IN) with and without adjuvant, the PBS+SQ group was used as control. The adjuvants used were squalene (SQ) and Freund's adjuvant (FA). Positive proliferation control was concanavalin A (ConA) and negative control Medium and BSA. All groups were compared against the PBS+SQ group. Data represent mean ± SD (n = 4). The *p* values were determined by Two-way ANOVA with Dunette post-tests. ns = not significant.
